# The BUDDY (Bedside Ultrasound to Detect Dehydration in Youth) study

**DOI:** 10.1186/s13089-014-0015-z

**Published:** 2014-09-10

**Authors:** Joshua Jauregui, Daniel Nelson, Esther Choo, Branden Stearns, Adam C Levine, Otto Liebmann, Sachita P Shah

**Affiliations:** 1 Department of Emergency Medicine, Warren Alpert Medical School, Brown University, Providence 02912, RI, USA; 2 Rhode Island Hospital, Providence 02903, RI, USA; 3 Division of Emergency Medicine, Harborview Medical Center, University of Washington, M/S 325 9th Avenue, Seattle 98104, WA, USA

**Keywords:** Ultrasound, Inferior vena cava, Aorta, Dehydration, Pediatric, Vomiting, Diarrhea

## Abstract

**Background:**

Prior research suggests that the ratio of the ultrasound-measured diameter of the inferior vena cava to the aorta correlates with the level of dehydration in children. This study was designed to externally validate this and to access the accuracy of the ultrasound measured inspiratory IVC collapse and physician gestalt to predict significant dehydration in children in the emergency department.

**Methods:**

We prospectively enrolled a non-consecutive cohort of children ≤18 years old. Patient weight, ultrasound measurements of the IVC and Ao, and physician gestalt were recorded. The percent weight change from presentation to discharge was used to calculate the degree of dehydration. A weight change of ≥5% was considered clinically significant dehydration. Receiver operating characteristic (ROC) curves were constructed for each of the ultrasound measurements and physician gestalt. Sensitivity (SN) and specificity (SP) were calculated based on previously established cutoff points of the IVC/Ao ratio (0.8), the IVC collapsibility index of 50%, and a new cut off point of IVC collapsibility index of 80% or greater. Intra-class correlation coefficients were calculated to assess the degree of inter-rater reliability between ultrasound observers.

**Results:**

Of 113 patients, 10.6% had significant dehydration. The IVC/Ao ratio had an area under the ROC curve (AUC) of 0.72 (95% CI 0.53 to 0.91) and, with a cutoff of 0.8, produced a SN of 67% and a SP of 71% for the diagnosis of significant dehydration. The IVC collapsibility index of 50% had an AUC of 0.58 (95% CI 0.44 to 0.72) and, with a cutoff of 80% collapsibility, produced a SN of 83% and a SP of 42%. The intra-class correlation coefficient was 0.83 for the IVC/Ao ratio and 0.70 for the IVC collapsibility. Physician gestalt had an AUC of 0.61 (95% CI 0.44 to 0.78) and, with a cutoff point of 5, produced a SN of 42% and a SP of 65%.

**Conclusions:**

The ultrasound-measured IVC/Ao ratio is a modest predictor of significant dehydration in children. The inspiratory IVC collapse and physician gestalt were poor predictors of the actual level of dehydration in this study.

## 1 Background

Acute volume depletion due to enteritis remains a common cause of morbidity and mortality in the pediatric population worldwide [1]-[4]. In the USA, acute diarrhea accounts for >1.5 million outpatient visits, 200,000 hospitalizations, and approximately 300 deaths/year [1]. Accurate assessment of the degree of dehydration in the emergency department (ED) remains a diagnostic challenge, but is crucial to the appropriate management of dehydrated children. The criterion standard used in research to determine degree of volume depletion is the percent weight change between the ill weight and the rehydrated or baseline weight of the patient, and it is a retrospective standard useful for clinical research [5],[6]. However, because the pre-illness weight is not available in the acute care setting, other parameters must be used to assess the degree of dehydration [7].

The diagnostic options available to clinicians hoping to discern mild from significant, or moderate or severe, dehydration include non-invasive measures such as physician gestalt and clinical scales derived from signs and symptoms, invasive hemodynamic monitoring, laboratory values, and bedside ultrasound techniques newly described in the literature. Unfortunately, clinical scales such as the World Health Organization (WHO) scale, the Gorelick scale, and the clinical dehydration scale (CDS) also known as the Parkin scale have been studied in a handful of trials, which show that none of these scales perform with a high measure of sensitivity to detect clinically significant dehydration in the pediatric emergency department population [8]-[15]. Similarly, lab values have limited sensitivity and specificity for the detection of acute dehydration [16],[17].

While there is often no need for invasive monitoring using central venous pressure (CVP) in pediatric patients presenting with dehydration, measurement of CVP until recently had been the invasive measure of choice to determine severity of volume depletion for patients with undifferentiated shock. Adult literature suggests CVP may not be a reliable indicator of volume depletion as the cause of hypotension and suggest CVP is unhelpful in predicting which patients will be ‘fluid responders’ and which patients may need other methods of blood pressure support [18],[19].

Bedside ultrasound has emerged as a potentially useful, non-invasive tool for the rapid assessment of degree of dehydration in both adults and children [17],[20]. Ultrasound measures of the inferior vena cava show that this thin-walled, collapsible vessel varies in size with respiratory changes in intra-thoracic pressure and overall diameter appears to change with overall blood volume. These features of the inferior vena cava (IVC) have allowed pioneering clinicians to measure both the collapsibility index and the ratio of the IVC to the thicker-walled aorta, which does not vary in size with the respiratory cycle or volume status of a patient.

In the pediatric population, there have been a few studies of bedside ultrasound of the IVC compared with the criterion standard of weight-change-determined degree of dehydration previously used in the literature. The ratio of the ratio of IVC and aorta diameters (Ao) (IVC/Ao) has been shown to correlate marginally well with the degree of volume depletion in children presenting with vomiting and diarrhea in these studies [17],[20].

Our study is the first ultrasound study to compare the criterion standard of greater than 5% weight change with rehydration to the collapsibility index of the IVC in children. It is also the first North American external validation of the IVC/Ao technique described by Chen et al. in a pediatric emergency setting [17],[21].

## 2 Methods

### 2.1 Study design

This was a prospective, non-consecutive cohort study conducted from June 2011 to February 2013. This study was approved by the Human Subjects Internal Review Board at Lifespan, Inc., and Rhode Island Hospital. Written informed consent was obtained by each subject's parent or legal guardian.

### 2.2 Setting and participants

The Bedside Ultrasound to Detect Dehydration in Youth (BUDDY) study was conducted in a busy, tertiary care pediatric emergency department (PED) at Hasbro Children's Hospital in Providence, Rhode Island, with an annual census of approximately 50,000 PED visits.

Subjects were enrolled as a convenience sample based on investigator availability. Investigators were available to enroll patients Monday through Friday, from 9 a.m. until 5 p.m., excluding holidays. All children less than or equal to 18 years of age presenting with a chief complaint of vomiting and/or diarrhea, or suspicion of dehydration by an attending pediatric emergency physician were eligible for enrollment. Eligible patients whose initial order sets included plans for intravenous fluids were approached for study consent, and those only receiving oral fluids were not included in the study. Exclusions criteria included positive pressure ventilation, significant traumatic injury, large volume fluid administration prior to enrollment, surgical abdomen, and known congenital cardiac disease or pulmonary hypertension. Only English speaking patients were enrolled.

### 2.3 Patient assessment

Enrollment, weights, and ultrasound exams of the subjects were conducted by trained research assistants and resident physicians in Emergency Medicine. All study staff participated in a brief training by the principle study investigator (SS) including technique for patient weights using infant and child scales and ultrasound technique for the two ultrasound measures recorded. Ultrasound training consisted of both a lecture (30 min) and hands-on practice with proctored exams (1 to 5).

### 2.4 Calculation of percentage of dehydration

Research assistants weighed each child prior to intravenous fluid administration and again at the completion of ED resuscitation. Children admitted to the inpatient service for further hydration were weighed again prior to hospital discharge. All weights were performed without the child wearing clothes and with the same calibrated study scale (Seca Iena 354 for infants and Seca 813 Robusta for children able to stand, Seca, Handover, MD). The final weight was recorded as the weight upon discharge from the ED or from the hospital for admitted patients. The initial weight was that obtained upon enrollment. The percent dehydration was determined using the following formula: (final weight − initial weight)/final weight × 100%. Subjects with a percentage weight change of 5% or greater were considered to be significantly dehydrated. Significant dehydration was defined as moderate (5 to 10%) and severe (>10%) dehydration combined as previously described in the pediatric literature [5],[10],[15],[17].

### 2.5 Ultrasound protocol

**Figure 1:**
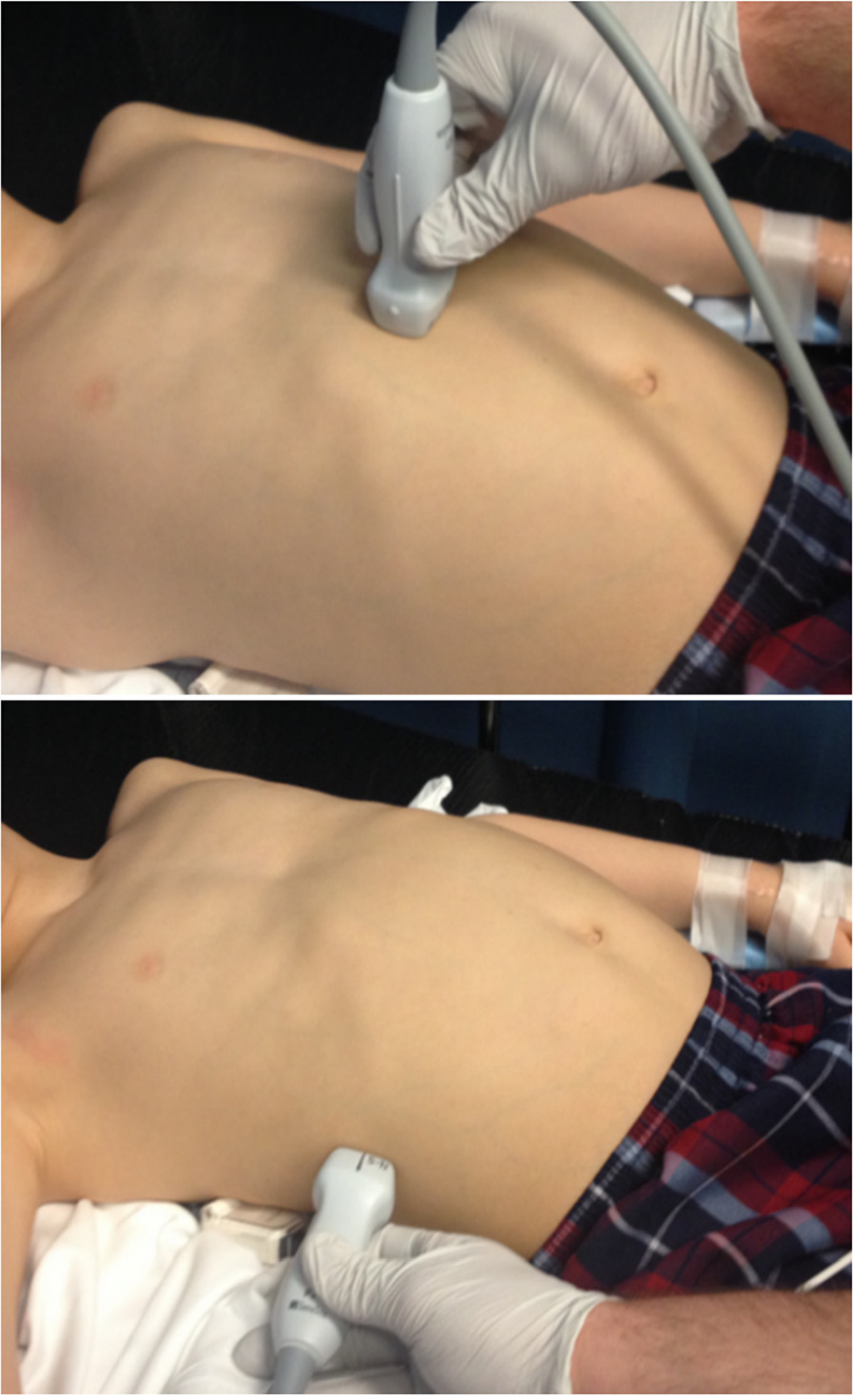
**Ultrasound probe positions to measure the IVC/Ao ratio in the transverse axis in the subxiphoid region above and to measure the IVC collapsibility in the longitudinal axis below.** Ao, aorta; IVC, inferior vena cava.

Bedside ultrasound was performed by a study investigator using a Sonosite M-Turbo ultrasound machine (Bothell, WA) and curved footprint C-60 abdominal probe or a phased array P21 probe at the time of enrollment (beginning of the ED visit). There were four sonographer investigators participating in the study. Investigators performing the ultrasound were aware of the inclusion criteria but blinded to the treating clinician's impression of the degree of dehydration. Treating clinicians were blinded to the ultrasound results. The two ultrasound techniques were performed with subjects in the supine position with measurements obtained in B mode (see Figure 1).

**Figure 2:**
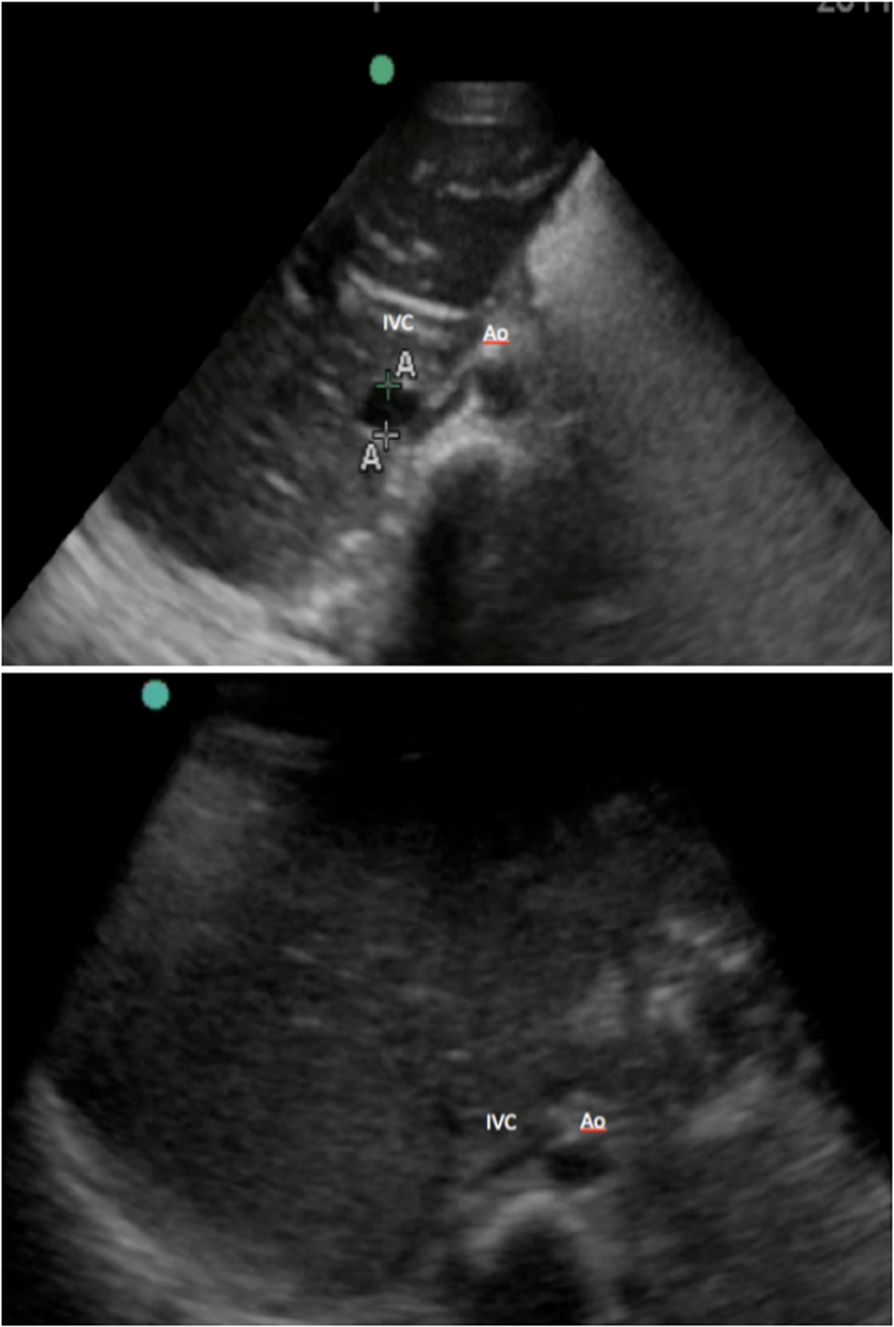
**Ultrasound image of the transverse inferior vena cava to aorta (IVC/Ao) diameter measurement in a child with an IVC/Ao ratio >0.8 (no dehydration) above and <0.8 below (significant dehydration).** Ao, aorta; IVC, inferior vena cava.

For the IVC/Ao ratio using a cross section of both vessels for measurements, the transducer was placed over the anterior abdomen in the axial or transverse plane just below the level of the xyphoid bone. The aorta and IVC were visualized in cross section, and a 6-s video clip in addition to measured still images were recorded for later review. Diameters of the Ao and IVC were both measured using calipers in their maximal size, during systole for the aorta, and expiration for the IVC, from anterior to posterior (see Figure 2).

**Figure 3:**
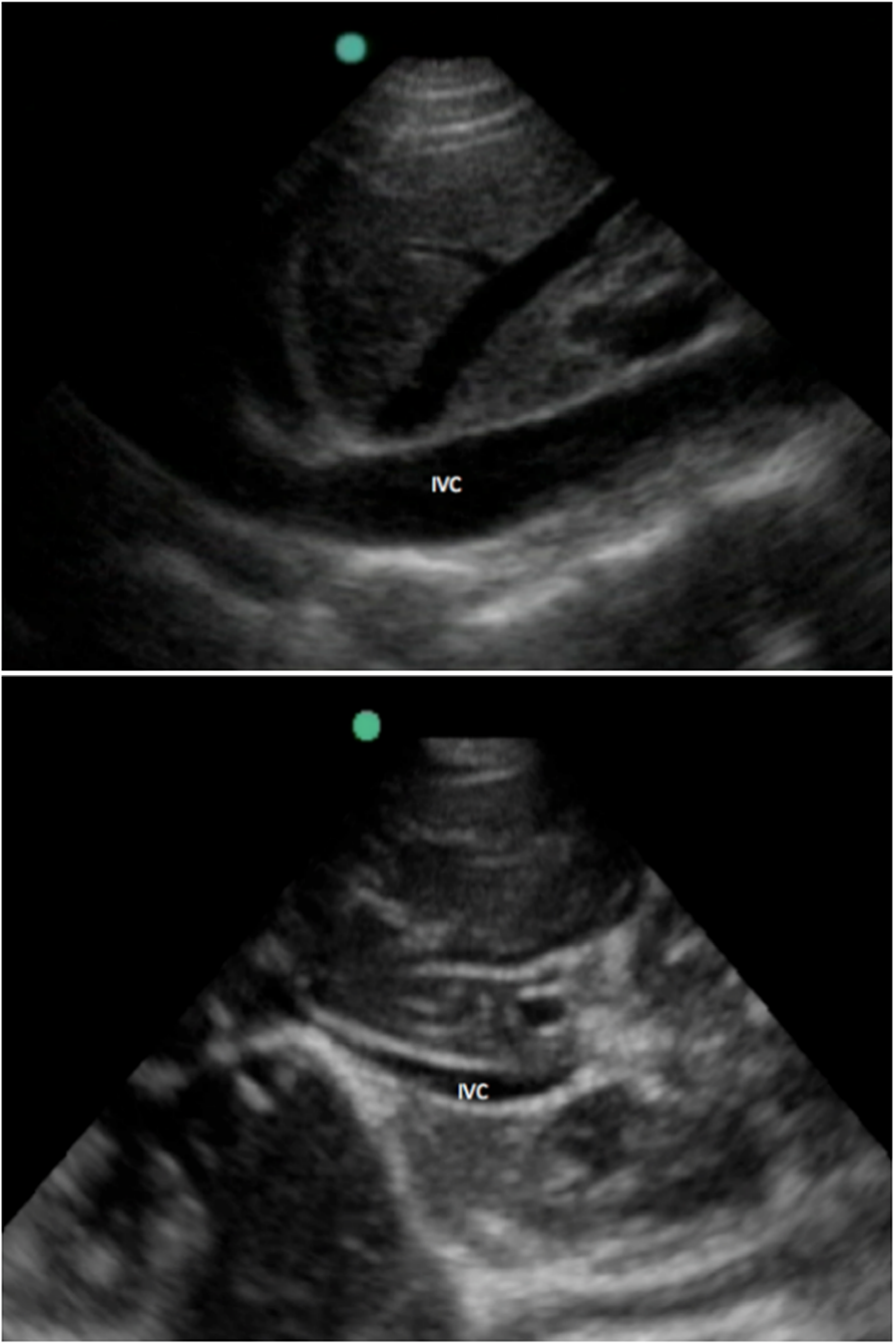
**Ultrasound image of the longitudinal inferior vena cava collapsibility in expiration above and inspiration below.** IVC, inferior vena cava.

For the long axis view of the IVC to assess collapsibility with inspiration, one of two techniques was used. The probe was either placed midline in the sagittal plane with the marker towards the patient's head or on the patient's right side, coronal plane, and mid-axillary line with the marker towards the head. The probe position was chosen based on where the study investigator felt the best view could be obtained using the two techniques previously described in the literature [22]-[25]. A 6-s video clip was recorded, and then still images during inspiration and expiration were obtained. Still images of the maximum and minimum diameter of the IVC were measured, 2 cm from the right atrium-caval junction at the diaphragm or near the level of the entry of the hepatic veins as described previously in the literature (see Figure 3) [26]-[28].

### 2.6 Data collection

After enrollment, the treating pediatric emergency medicine attending physician, who had examined the patient and was blind to the ultrasound findings, recorded their clinical gestalt for percent dehydration on a data sheet with a 1 to 10 scale of gestalt of dehydration. Afterward, a research assistant documented the patient's vitals, weight, clinical score, and volume of fluid administered.

### 2.7 Outcomes

Our primary outcomes were to assess the accuracy of ultrasound measurements of the IVC/Ao ratio and the collapsibility index of the IVC, for prediction of significant dehydration in children relative to the criterion standard of percent weight change. Our secondary outcome was to assess the accuracy of physician gestalt compared to the same criterion standard.

### 2.8 Data analysis

First, we calculated basic population demographics using descriptive statistics. We then constructed receiver operating characteristic (ROC) curves to evaluate the accuracy of the IVC/Ao ratio, collapsibility index of the IVC, and physician gestalt compared to our criterion standard. We also calculated the sensitivity, specificity, likelihood ratio positive (LR+), and likelihood ratio negative (LR−) for both ultrasound measurements and physician gestalt. We calculated these test characteristics using the traditionally used cutoff points of IVC/Ao ratio (0.8) and collapsibility index of 50%. In addition, we calculated test characteristics for a cutoff point of IVC collapsibility index of 80% or greater because it was the best cutoff point obtained on the ROC curve to rule out dehydration and, therefore, the most clinically relevant.

To determine inter-rater reliability between ultrasound observers, an ultrasound fellowship trained investigator, blinded to the study results and prior measurements, reviewed the ultrasound clips of a selected random subgroup of 30 patients, and re-measured the IVC/Ao ratio, as well as the maximum and minimum diameter of the long axis view of the IVC during the normal breath cycle. Intra-class correlation coefficients were calculated to determine the degree of reliability for these measurements.

Statistical analyses were performed using Stata version 11.0 (StataCorp LP, College Station, TX).

### 2.9 Sample size

The sample size was originally calculated based on the IVC/Ao ultrasound component of the study. Using standard algorithms from the literature, an area under the ROC curve (AUC) of 0.756 for the performance of IVC ultrasound as a predictor of dehydration based on data previously obtained, a type I error rate of 0.05, a type II error rate of 0.20, and the proportion of children with significant (>5%) dehydration, in prior studies conducted in North American hospitals, we determined we would need to enroll at least 112 children to be sure that the 95% confidence intervals for our ROC curve did not cross the reference line (or null hypothesis). Significant dehydration was defined as moderate (5 to 10%) and severe (>10%) dehydration combined as previously described in the literature [5],[10],[15],[17].

## 3 Results

### 3.1 Demographics

We approached 209 patients for potential enrollment, 61 declined to consent. Of the 148 remaining patients, 35 withdrew from the study or were excluded prior to completion of data collection, leaving 113 children for analysis. No patients were excluded because of poor diagnostic window. Of the 113 enrolled children, 39 were admitted and 74 were discharged from the ED with 58 male and 55 female. Median age for the patients enrolled was 6 years (range 1 month - 18 years). Twenty-nine children were under 3 years of age and 49 were under 5 years of age. Basic demographics with corresponding *P* values of the final subjects are described in Table 1. *P* values were two-sided, and a *P* value lower than 0.05 was considered as statistically significant.

**Table 1:** Demographics of children

**Demographic variable**	**All patients**	**Patients with significant dehydration (≥5%)**	**Patients without significant dehydration (<5% dehydration)**	***P*** **value**^**a**^
Median age, years (range)	6 (1 month-18 years)	2.5 (6 months to 15 years)	7 (1 month to 18 years)	0.05^b^
Median fluid administered (cc/kg)	21	29	20	0.004^c^
Admitted (%)	39 (34.5%)	7 (58.3%)	32 (31.7%)	0.07^d^
Less than 5 years old (%)	49 (43.4%)	9 (75%)	40 (39.6%)	0.02^d^
Total children (%)	*n* = 113	*n* = 101 (89.4%)	*n* = 12 (10.6%)	

^a^Comparing patients with and without significant dehydration. ^b^Two-tailed *t* test. ^c^Two-sample Wilcoxon ranksum. ^d^Two-tailed chi-square test.

### 3.2 Outcomes

Volumes of fluid resuscitation were determined clinically by the treating clinician and ranged from less than 20 cc/kg in 34% of patients, greater than 20 cc/kg but less than 40 cc/kg in 52% of patients, and greater than 40 cc/kg in 14% of patients. The average percent weight change with rehydration was 2.8%. Twelve patients (10.6%) had significant dehydration, defined as a percentage weight change greater than 5% with rehydration. Of these 12, five patients had a percentage weight change greater than 10%.

**Figure 4:**
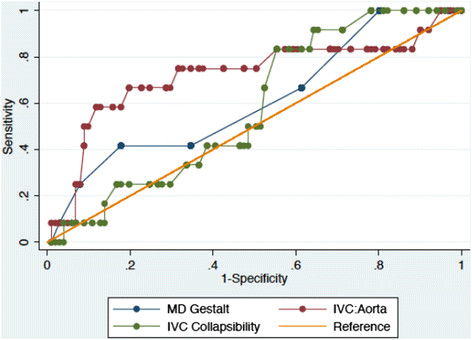
**Receiver operating characteristic curves for the ultrasound measured inferior vena cava to aorta ratio, the ultrasound measured inspiratory inferior vena cava collapsibility, and physician gestalt.** IVC/Aorta, inferior vena cava to aorta ratio; IVC, inferior vena cava; MD, physician.

Test characteristics for the two ultrasound measurement techniques and physician gestalt are shown in Table 2 and comparison of the ROC curves for each ultrasound measurement technique and gestalt are demonstrated in Figure 4.

**Table 2:** Test characteristics for the ultrasound measured inferior vena cava to aorta ratio diameter, the ultrasound measured inferior vena cava inspiratory collapse, and physician gestalt

**Technique (cut point)**	**AUC (95%CI)**	**SN (%)**	**SP (%)**	**LR+**	**LR**−
IVC/Ao US (0.8)	0.72 (0.53, 0.91)	67	71	2.32	0.47
Inspiratory collapse of IVC US (80%)	0.58 (0.44, 0.72)	83	42	1.43	0.40
Physician gestalt (≥5)	0.61 (0.44, 0.78)	42	65	1.20	0.89

AUC, area under the receiver operating characteristic curve; SN, sensitivity; SP, specificity; LR+, likelihood ratio positive; LR−, likelihood ratio negative; IVC/Ao, inferior vena cava to aorta ratio; IVC, inferior vena cava; US, ultrasound.

### 3.3 Accuracy

The IVC/Ao ratio had an area under the curve (AUC) that was statistically significantly different from the reference line. The AUC was 0.72 (95% CI 0.53 to 0.91) with SN of 67% and SP of 71% for prediction of significant dehydration when using the previously established cutoff point of 0.8. The positive likelihood ratio (LR+) for this exam was 2.32.

The IVC collapsibility index had an AUC of 0.58 (95% CI 0.44 to 0.72), which was not statistically significant compared with the reference line. A cutoff of 50% collapsibility, which has been previously established in the adult literature, produced a SN of 8% and a SP of 87%, with LR + 0.65. However, a cutoff of 80% collapsibility produced a SN of 83%, a SP of 42%, and a LR + 1.43 for the prediction of significant dehydration.

Physician gestalt had an AUC of 0.61 (95% CI 0.44 to 0.78), which was also not statistically significantly different from the reference line. A cutoff point of 5, which correlated with moderate or severe dehydration as labeled on the Likert scale physicians used to rate dehydration, produced a SN of 42%, SP of 65% and a LR + 1.20 for prediction of significant volume depletion.

### 3.4 Reliability

In a subgroup of 30 patients, the IVC/Ao ratio measurement had an intra-class correlation coefficient of 0.83 (95% CI 0.63 to 0.92) and the IVC collapsibility measurement had an intra-class correlation coefficient of 0.70 (95% CI 0.35 to 0.86).

## 4 Discussion

Acute dehydration in children is a common condition with potentially severe morbidity and mortality. Accurate assessment of the degree of dehydration can help clinicians guide treatment with either oral or intravenous fluid resuscitation, and is necessary for accurate prognosis and resource management. In the literature, the established gold standard for assessing the degree of dehydration in children is the percent weight change between the pre-illness weight and the acute illness weight of the child [5],[10],[15],[17]. Simply put, it is the weight the child loses due to the illness. Because the pre-illness weight is not available in the acute care setting, other parameters must be used to assess the degree of dehydration in children. However, historical, physical, and laboratory findings lack the sensitivity, specificity, and reliability to be helpful in differentiating children with significant dehydration [5].

Prior research suggests that the ratio between the ultrasound measured diameter of the IVC and the aorta correlates with the level of dehydration in children with good inter-rater reliability [17],[21]. In this study, the IVC/Ao ratio had an AUC that was statistically significant from the reference line, and therefore, the use of ultrasound to measure the IVC/Ao ratio for detecting dehydration in children now has external validity evidence for being an objective adjunct in the assessment of dehydration in children.

On 14 October 2013, the American College of Emergency Physicians (ACEP) announced its list of five recommendations as parts of the ‘Choosing Wisely’ campaign in an effort to use appropriate tests and treatments in patients and to avoid care when the harm outweighs the benefit. One of the recommendations was the following: ‘Avoid instituting intravenous (IV) fluids before doing a trial of oral rehydration therapy in uncomplicated emergency department cases of mild to moderate dehydration in children’ [29]. As such, in order for appropriate application of this recommendation, it is important to determine the degree of dehydration prospectively. We believe that the ultrasound-measured IVC/Ao diameter may be a helpful tool in differentiating among children with varying degrees of dehydration, especially given its limited cost and ease of availability.

Significant inspiratory IVC collapse, as measured by ultrasound, has been shown to be helpful in the assessment of volume status in adults [30],[31]. Inspiratory IVC collapse has been shown not to correlate with central venous pressure (CVP) measurements in children admitted to a pediatric critical care unit in children [32]. However, the utility of CVP has come into question and the gold standard for assessing dehydration in children is well established in the literature to be percent weight change [5],[10],[15],[17]-[19]. This study adds to the literature by demonstrating that the inspiratory IVC collapse did not have an AUC statistically significantly different from the reference line when compared with the gold standard of percent weight change and, therefore, was no better than chance at predicting significant dehydration in our cohort of children presenting to the PED. This study would suggest that there is little utility to using the ultrasound-measured collapsibility of the IVC in the assessment of children with possible dehydration. However, this may have been due to the technical difficulty inherent to the measurement in children who are crying and have respiratory patterns that are difficult to assess. In crying or actively ‘sniffing’ children, there is breath to breath variation in tidal volume which significantly affects inspiratory collapse of the IVC and ability to visualize the diaphragm which is needed for accurate assessment of the IVC.

Other possibilities that may have contributed to the poor performance of respiratory variation in IVC size in children when measured in long axis are technical in nature. A recent study of ultrasound measures of IVC diameter suggests greater variability in both cranio-caudal and medio-lateral movements of the vessel relative to the transducer with breathing motion in the long axis view of the IVC compared with the short axis technique [33]. Cranio-caudal movement of the vessel during the respiratory cycle may lead to comparison of different portions of the IVC during inspiration and expiration, but even more worrisome is the medio-lateral movement of the cylindrical vessel relative to the probe, which can lead to off midline diameter measurements and subsequent decreased accuracy in assessment of diameter. Further research is warranted in patients with volume depletion to determine what role these factors play in overall accuracy of IVC measurement as a tool for detection of dehydration.

Although physicians historically have estimated the degree of dehydration based on clinical gestalt, this study confirms the findings of previous evidence that physician gestalt is a poor predictor of significant dehydration in children.

### 4.1 Limitations

This was a prospective, non-consecutive cohort study; children were enrolled based on research assistant availability, which limited our overall pool of patients approached. We included physician gestalt because this is frequently used in the clinical setting to assess dehydration; however, the study was not specifically powered to demonstrate the superiority of ultrasound over physician gestalt. The study was powered to show whether each of the ultrasound exams was statistically better than chance to determine significant dehydration. In addition, we excluded patients who had already received a significant volume of intravenous fluids, which likely includes the most acutely ill children receiving an immediate intravenous line and fluids on arrival in dedicated resuscitation rooms.

When calculating the percent weight change, we used the final, post-rehydration weight as a surrogate marker for the pre-illness weight. As such, we assume the ED discharge weight and the hospital discharge weight reflect the pre-illness weight. While recent research has found post-rehydration weight correlates almost perfectly with pre-illness weight, one study demonstrated that post-rehydration weight tends to underestimate pre-illness weight [34].

Finally, we only assessed inter-rater reliability for the ultrasound measurements in a randomly chosen subset of patients in this study for quality assurance. Inter-rater reliability of the creation of the ultrasound images as well as physician gestalt was not assessed, and it is possible that there may be significant variability among the gestalt that clinicians have when assessing dehydration in patients. We attempted to mitigate this by only allowing pediatric emergency medicine-trained attending physicians to determine the gestalt of the patient's level of dehydration for our study.

## 5 Conclusions

In this study, the IVC/Ao ratio, as measured by bedside ultrasound, is a modest predictor of significant dehydration in children. The inspiratory IVC collapse, as measured by bedside ultrasound, and physician gestalt were not helpful in assessing the degree of dehydration in our cohort.

## Abbreviations

IVC: inferior vena cava

Ao: aorta

IVC/Ao: ratio of inferior vena cava and aorta diameter

IVC: intravenous

ROC: receiver operating characteristics curve

SN: sensitivity

SP: specificity

AUC: area under the receiver operating characteristic curve

CI: confidence interval

ED: emergency department

WHO: World Health Organization

CDS: clinical dehydration scale

CVP: central venous pressure

LR+: likelihood ratio positive

LR−: likelihood ratio negative

ACEP: American College of Emergency Physicians

## Competing interests

The authors declare they have no competing interests.

## Authors' contributions

JJ helped conceptualize and design the study, supervised collection of data, drafted the initial manuscript, and approved the final manuscript as submitted. DN helped conceptualize and design the study, supervised collection of the data, and approved the final manuscript as submitted. EC carried out the statistical analysis, reviewed and revised the manuscript, and approved the final manuscript as submitted. BS oversaw data collection and approved the final manuscript as submitted. AL provided guidance for study design and content expertise, reviewed and revised the manuscript, and approved the final manuscript as submitted. OL aided in study design and approved the final manuscript as submitted. SS oversaw the study, reviewed and revised the manuscript, and approved the final manuscript as submitted. All authors read and approved the final manuscript.

## Authors' information

JJ is a senior medical education fellow/acting instructor of emergency medicine at the University of Washington in Seattle, WA, USA, specializing in the training and education of residents and students in ultrasound. EC is an assistant professor of emergency medicine at Brown University in Providence, RI, USA, with fellowship training in policy and research in emergency medicine. AL is an assistant professor of emergency medicine and director of the global emergency medicine fellowship at Brown University in Providence, RI, USA. OL is an assistant professor and director of the division of emergency ultrasound at Brown University in Providence, RI, USA. SS is an assistant professor of emergency medicine at the University of Washington in Seattle, WA, USA, with fellowship training in emergency ultrasound. She specializes in teaching ultrasound in resource limited settings.
